# Effects of Polyphenolic Extracts From Sumac, Pomegranate Peel, Indian Almond Leaves, Falsa, and Banana Bracts on Calcium Oxalate and Brushite Crystallization In Vitro

**DOI:** 10.1002/cbdv.202500023

**Published:** 2025-04-08

**Authors:** Mudassir Nazir, Muhammad Abdul Haq, Syeda Moahida Batool Sherazi, Shahina Naz, Lubna Mobin, Alexandros Tsoupras

**Affiliations:** ^1^ Department of Food Science & Technology University of Karachi Karachi Sindh Pakistan; ^2^ Department of Chemical Engineering and Applied Chemistry University of Toronto Toronto Canada; ^3^ Hephaestus Laboratory, School of Chemistry Faculty of Science, Democritus University of Thrace, University of Thrace, Kavala University Campus St Lukas Kavala Greece

**Keywords:** anthocyanins, antilithogenic, brushite crystals, calcium oxalate, food bioactives, non‐anthocyanins, polyphenols, valorization

## Abstract

Nephrolithiasis, or kidney stone formation, is a widespread global health concern. This study examines the effects of polyphenolic extracts, along with their anthocyanin and non‐anthocyanin fractions, from sumac fruit, pomegranate peel, almond leaves, falsa fruit, and banana bracts on the crystallization of calcium oxalate (CaC_2_O_4_·nH_2_O) and brushite (CaHPO_4_·2H_2_O) in vitro. The extracts were prepared through maceration in aqueous methanol and further fractionated into anthocyanin and non‐anthocyanin fractions using solid‐phase extraction. For calcium oxalate crystallization, nucleation and aggregation were monitored using a spectrophotometer in the presence and absence of these extracts and fractions. For brushite crystallization, the single diffusion gel growth method was employed. All extracts inhibited the crystallization of both calcium oxalate and brushite in a dose‐dependent manner, significantly reducing crystal number, size, and altering crystal morphology. Non‐anthocyanin fractions demonstrated a stronger inhibitory effect than anthocyanin fractions. Molecular docking studies further revealed that compounds in these fractions exhibited strong binding affinity with proteins involved in the adhesion and aggregation of calcium oxalate crystals to renal cells, supporting their antilithogenic properties. These findings suggest that these natural polyphenolic sources hold promise as potential inhibitors of kidney stone formation.

## Graphical abstract

Polyphenolic extracts and their fractions from five plant sources inhibit calcium oxalate and brushite crystal formation in vitro, with non‐anthocyanin fractions showing stronger anti‐lithogenic effects supported by molecular docking.

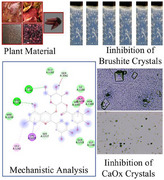

## 1 Introduction

Nephrolithiasis (kidney stone formation) is a highly prevalent disease with a recurrence rate of more than 50% in all communities irrespective of age, gender, culture, and race [1, 2]. The incidence of kidney stones is increasing globally; an increase in deaths from 0.81 million in 2000 to 1.3 million in 2019 due to kidney failure has been estimated [3]. Multiple factors are responsible for stone formation and growth. In one third of patients, the main reason is family history or genetic susceptibility [4, 5], whereas in the remaining two‐thirds of the cases, environmental and behavioral factors, particularly food, are responsible for this. Among all the types of kidney stones, calcium stones have the highest occurrence rate, and for the subtypes of calcium stones, the order of occurrence is calcium oxalate [CaC_2_O_4_·nH_2_O] (80%)> calcium phosphate [apatite, Ca_10_(PO_4_)6(OH_2_)] (4%–6%)> calcium hydrogen phosphate dihydrate [brushite, CaHPO_4_·2H_2_O] (CHPD) (2%–6%). Severity of renal pathological damage caused by stones depends on their chemical nature and morphological features. For example, brushite stones cause more severe renal damage and are harder to comminute compared to apatite and calcium oxalate; brushite removal often requires surgery [6]. Similarly, calcium oxalate monohydrate (CaC_2_O_4_·H_2_O) crystals compared to calcium oxalate dehydrate (CaC_2_O_4_·2H_2_O) are reported to be more potent in nephrolithiasis owing to their higher affinity for renal cells [7]. Moreover, CaC_2_O_4_·H_2_O is thermodynamically a more stable form [8]. Irrespective of the type, the process of stone formation passes through two distinct stages of nucleation and aggregation. Nucleation, which is usually triggered by high concentration of lithogenic ions, results in small crystals in renal tubules, whereas in aggregation, small crystals unite to form aggregates [9, 10, 11, 12]. Inside the renal stone matrix, various proteins are present that support the adhesion and interaction of crystals to membranes. Among these proteins, five have already been isolated from human renal stones and identified as ethanolamine‐phosphate cytidylyltransferase, Ras GTPase‐activating‐like protein, UDP‐glucose glycoprotein glycosyltransferase 2, RIMS‐binding protein 3A, and macrophage‐capping protein [13].

Stone formation is appreciably affected by adequate hydration, a low intake of animal proteins, lithogenic compounds (such as oxalate and purines), and a diet rich in antilithiatic compounds such as citrate [14]. Thus, one of the ways to seize initiation of stone formation is to avoid supersaturation of stone‐forming components in urine [15] by using antilithogenic substances. However, once crystals are grown to large size, they are removed by some surgical procedures [16]. Even after surgical removal, the recurrence of stones [17] remains a constant threat for patients, and some have to go through multiple surgeries. Thus, there is a need for natural and safer remedies like herbs or plant food to control this recurrence. Several studies proved that kidney stones can be prevented by intake of diet rich in polyphenols. Dietary plants, like green tea, pomegranate, oregano, parsley, raspberry, black cumin, orange, lemon, lime, and grapefruit, have been found to be significantly effective in treating lithogenesis [18]. With more than 8000 structural variations, polyphenols display significant activities against the oxidative stress‐related kidney dysfunction and formation of renal stones [19, 20, 21, 22]. Chaudhary et al. demonstrated the potential of *Terminalia arjuna* bark to inhibit the formation of both CHPD and CaC_2_O_4_·nH_2_O crystals in vitro [23]. Similarly, Sharma et al. concluded from their study that the leaves of *Chenopodium album* could inhibit in vitro crystallization of CaC_2_O_4_·nH_2_O and CHPD [24]. Green tea polyphenols have been found to favor the CaC_2_O_4_·2H_2_O crystal formation over CaC_2_O_4_·H_2_O [25], and the role of these polyphenols as step pinners or kink blockers or as both in suppression of CaC_2_O_4_·H_2_O crystallization has been mechanistically proved [8]. The role of polyphenolics has not only been demonstrated in crystal inhibition but also in protein‐assisted crystal adhesion with membranes. Molecular docking studies of 35 compounds selected from *Enydra fluctuans* species (plant traditionally used for the treatment of kidney stones in India and Bangladesh) with 5 stone matrix‐associated proteins revealed that only 2 molecules that had satisfactory docking scores were flavonoids. [26]

Being powerful antioxidant, phyto‐phenols are quite effective against nephrolithiasis; therefore, it is worthwhile to carry out detailed research on the plants that have been traditionally used in management of kidney stones and/or have modern experimental evidence of anti‐urolithiatic activity but have never been fully explored in this regard. The aim of this study is to evaluate the inhibitory effects of polyphenolic extracts from sumac fruit, pomegranate peel, almond leaves, falsa fruit, and banana bracts, along with their anthocyanin and non‐anthocyanin fractions, on the crystallization of calcium oxalate and brushite in vitro. Fruits of *Rhus typhina* (sumac) (SF), *Grewia asiatica* (falsa) (FF), *Punica granatum* (pomegranate) peel (PP), leaves of *Terminalia catappa* (red almond) (AL), and bracts of *Musa paradisiaca* (banana bract) (BB) are very well known for their unique polyphenolic composition [27, 28, 29, 30, 31, 32] and have been explored for various therapeutic applications. In one of our recent research study [33], we have demonstrated the inhibitory effect of crude methanolic extracts, anthocyanin, and non‐anthocyanin fractions of SF, PP, and AL on the growth of urease‐producing species and jack bean urease activity.

Besides, being unique in their composition and therapeutic potential, these polyphenolic sources are either underutilized or are plant waste materials that further justify their selection for the study. Though some studies have reported CaC_2_O_4_·nH_2_O anti‐crystallization effect of banana stem [34], falsa leaves [35], and tree bark [23]. None of these studies evaluated inhibition activities of banana bract, falsa and sumac fruits, and almond leaves. However, anti‐crystallization effect of hydroalcoholic extract of pomegranate peel on calcium oxalate has been reported by Kachkoul et al. [36]. This is the first research study that compares the relative efficacy of crude extract, anthocyanin, and non‐anthocyanin fractions in inhibiting both the nucleation and aggregation of crystals. The study seeks to assess how these extracts influence crystal nucleation, aggregation, and morphology and to determine their potential as natural inhibitors of kidney stone formation by examining their binding affinity with proteins involved in crystal adhesion to renal cells.

The crude polyphenolic extracts (Exs: SFEx, FFEx, PPEx, ALEx, BBEx) were fractionated on HLB cartridges into anthocyanin (AFrs: SFAFr, FFAFr, PPAFr, ALAFr, BBAFr) and non‐anthocyanin fractions (NFrs: SFNFr, FFNFr, PPNFr, ALNFr, BBNFr) that allowed the removal of sugars and acids to determine the antilithogenic effect merely due to polyphenols. The effect of Exs and fractions (Frs) on % reduction in nucleation (%Ni) and aggregation (%Ai) of CaC_2_O_4_·nH_2_O was followed spectrophotometrically, whereas the number, size, and morphology of crystals were determined by light microscopy. The effect on growth of CHPD was followed by single diffusion gel growth method. In addition, proteins isolated and identified in stone matrix in previous studies were docked with selected compounds present in the Frs to determine their role, if any, in the protein‐assisted crystal‐membrane interaction and adhesion. Up to 90% inhibition of nucleation and aggregation was observed that is comparable with previous studies on other plant extracts [37, 38, 39].

## 2 Materials and Methods

### 2.1 Collection and Selection of Raw Material

The bracts of banana and almond leaves were collected from the nursery of University of Karachi in the months of November and December. Sumac and falsa were purchased from the local market during the midsummer season, whereas pomegranate peel was collected in December. The plant materials were identified by Dr. Muneeba Khan, Taxonomist, at the Herbarium, University of Karachi. The identified species include *G. asiatica* (Voucher No. 99773), *T. catappa* (Voucher No. 99774), *M. paradisiaca* (Voucher No. 99775), *P. granatum* (Voucher No. 99776), and *R. typhina* (S. No. 448). The specimens have been deposited in the herbarium for reference. All plant samples were sorted, washed, packed, and stored in freezer at −15°C. All solvents and chemicals, including calcium chloride (CaCl_2_), calcium oxalate (Na_2_C_2_O_4_), sodium metasilicate (Na_2_SiO_3_), and orthophosphoric acid (H_3_PO_4_), were analytical grade and purchased from either BDH or Merck. Oasis HLB 6 cc Vac cartridges, with 200 mg sorbent (made from a co‐polymer of divinylbenzene and vinyl pyrrolidinone, Waters, USA), were used for separating anthocyanin fraction from non‐anthocyanins.

### 2.2 Extraction and Fractionation of Polyphenols

Following procurement, all plant samples were washed, freeze dried, and then extracted with acidified methanol. In a typical extraction procedure, 200 g of plant material were placed in 2 L of 80% (v/v) aqueous methanol containing 0.01% (v/v) HCl in amber glass bottles. The bottles were then placed in a shaking water bath for 2 h. Afterward, the mixture was filtered through Whatman No. 2 filter paper using a Buchner funnel under vacuum suction. The residue was then re‐extracted with fresh acidified aqueous methanol, and the process was repeated twice, for a total of three extractions. The combined extracts were pooled, and the solvent was removed using a rotary evaporator (Butchi, Rotavapor R‐100, Switzerland) at 35°C under vacuum. Any remaining solvent and water traces were removed using a freeze dryer (EYELA FD‐1000, Japan), and yield was calculated by weighing the freeze‐dried material (Table S1). Simple extraction was preferred over ultrasonic‐assisted or Soxhlet extractions as some polyphenols are highly susceptible to thermal treatment and sonochemical oxidation and degradation [40]. Reason for choosing acidified methanol as a solvent is because of its efficiency in extracting polar compounds, improving extraction yield by increasing the solubility of the target compounds, and stabilizing the free radicals that could possibly be generated during extraction [41, 42].

The dried samples were fractioned into AFrs and NFrs by applying the procedure of Kim and Lee [43]. The fractionation was performed using solid‐phase extraction vacuum manifold (Fisher Scientific SPE‐FTSPEMF12G USA). Ten milliliters of ethyl acetate, pure methanol, and 10 mM aqueous HCl were consecutively eluted through the cartridges in order to precondition the HLB columns. The crude phenolic extracts were first filtered using 0.45 µm syringe filter and then loaded onto the columns, followed by an additional 10 mL of 10 mM aqueous HCl to remove sugars, acids, and other water‐soluble substances. To elute the non‐anthocyanin fractions, the columns were first dried by‐passing nitrogen through them for at least 30 min, then eluted with 15 mL of ethyl acetate. Subsequently, 10 mL of acidified methanol (containing 0.01% HCl) was used to elute the remaining anthocyanin fractions. The solvents from these fractions were evaporated at 30°C (under low pressure), and the dried extracts were placed at −4°C until use. Total phenol and total anthocyanin were measured using Folin–Ciocalteu assay [44] and pH differential method, respectively (Table S1) [45].

### 2.3 Effects of Polyphenolic Extracts on In Vitro Crystallization

#### 2.3.1 In Vitro Crystallization of Calcium Oxalate

Nucleation and aggregation assays were performed according to the method de‐scribed by Hess et al. and adapted by Mittal et al. [46, 47]. With some modifications, that is, no stirring was performed throughout the experiment. Freshly prepared solutions of 10.0 mM calcium chloride (CaCl_2_) and 1.0 mM sodium oxalate (Na_2_C_2_O_4_), containing 200 mM sodium chloride (NaCl) and 10 mM sodium acetate, were adjusted to pH 5.7 and then warmed up to 37°C. For the measurement of crystallization in the absence of sample, 950 µL of CaCl_2_, 950 µL of Na_2_C_2_O_4_, and 100 µL water were mixed in a cuvette to record optical density at 620 nm after every 1 s over 20 min using spectrophotometer (Cary 60 UV–Vis, Agilent, USA). To determine the effect of Exs and Frs, 100 µL of each sample in varying concentrations (2, 4, and 8 mg/mL in water) was mixed with 950 µL of both CaCl_2_ and Na_2_C_2_O_4_ solutions, and optical density was recorded as above. Slopes of nucleation and aggregation phases were calculated using linear regression in MS Excel from the data curves generated, and % reduction in nucleation (%Ni) and aggregation (%Ai) in the presence of samples was calculated as (1‐SN*_i_*/SN*_c_*) × 100 and (1‐SA*_i_*/SA*_c_*) × 100, respectively, where SN*_c_* = nucleation slope with control, SN*_i_* = nucleation slope with inhibitor, SA*_c_* = aggregation slope with control, and SA*_i_* = aggregation slope with inhibitor (Figure S1). The tested concentrations (4, 2, and 1 mg/mL) were selected based on preliminary in vitro anti‐crystallization trials. The highest concentration (4 mg/mL) was chosen because it demonstrated >90% inhibition for certain fractions. To evaluate dose dependency, concentrations were sequentially reduced to half (2 mg/mL) and one‐fourth (1 mg/mL) of the initial concentration.

#### 2.3.2 Microscopic Analysis of Calcium Oxalate Crystals

The crystals were observed under a microscope (Optika, Italy) in the presence and absence of the extracts using the procedure of De Bellis et al. [7]. In small glass vials, 475 µL of the CaCl_2_ solution, 50 µL of test sample (4 mg/mL), and 475 µL of the Na_2_C_2_O_4_ solution were added sequentially. A vial containing 50 µL of distilled water instead of sample was used as the reference control. The contents were left in the vials for 20 min, and then 10 µL portion from each vial was drawn after shaking and spread uniformly over an area of 1 mm on a clean glass slide. Slides were left at room temperature and then analyzed next day by microscope at different magnifications in terms of crystal size, shape, and abundance. Microscopic analysis covered the entire area over which the sample was spread, and images were captured at 400× for multiple fields of view and processed by ImageJ for the number and size distribution of crystals.

#### 2.3.3 In Vitro Dissolution of Brushite (CHPD) Crystals

Brushite crystallization assay was carried out by single diffusion gel growth method as described by Sharma et al. [24]. Five hundred milliliters of sodium metasilicate solution (specific gravity 1.06) were acidified by adding 270 mL of orthophosphoric acid to obtain a mixture of pH 5.0. A portion of 7.5 mL of the mixture was then transferred to glass test tubes (2.5 cm diameter and 15 cm length) that were allowed to stand till formation of gel. After gel setting, crystals were grown in the gel by carefully pouring 10 mL of 1 M calcium chloride on the set gels. Crystals were allowed to grow for 5 days, and their apparent length was measured and recorded. A volume of 10 mL of aqueous solutions of samples (in 2 different concentrations of 4 and 8 mg/mL) were then added on fifth day, and their effect was studied on the growth of crystals up to the eighth day in terms of number and length. The results were compared with the control tubes in which distilled water was added instead of sample.

#### 2.3.4 In Vitro Inhibition of Brushite Crystals

For the inhibitory effect of the Exs and Frs on the formation of crystals, the above‐mentioned procedure was followed with some changes. Here, the sample extract and calcium chloride were added at the same time after gel formation to allow the inhibitor compounds to hinder the formation and growth of crystals compared to the one in which only calcium chloride was added.

#### 2.3.5 Docking in AutoDock Vina

PyRx and Discovery Studio were used for docking experiments and visualization between selected ligands and binding proteins. PyRx is a combination of different virtual screening tools [48]. This application uses Python as the programming language, Open Babel to import SDF files, and AutoDock Vina for actual docking. The conformation with the highest binding affinity was chosen for additional examination among the nine con‐formations that each ligand formed during the autodocking process. Discovery Studio was used to do post‐docking evaluations, which included thorough ligand–receptor interactions in both 2D and 3D forms.

#### 2.3.6 Ligand Selection and Preparation

For the docking process, the primary polyphenolic compounds previously identified in various extracts, as detailed in Table S2, were selected. The structures of these compounds were sourced from the PubChem website and downloaded accordingly. Each structure was then opened in Open Babel, where their geometry was optimized, and energy minimized. The optimized structures were saved in PDBQT format for subsequent docking using AutoDock Vina.

The 3D structures of ethanolamine‐phosphate cytidylyl transferase (PDB ID: 3ELB), macrophage‐capping protein (PDB ID: 1J72), and RasGTPase‐activating‐like protein (PDB ID: 3FAY) were obtained in PDB format from RCSB Protein Data Bank, whereas the PDB files of UDP glucose: glycoprotein glucosyl transferase 2 (Gene: UGGT2) and RIMS‐binding protein 3A (Gene: RIMBP3) were obtained from AlphaFold Protein Structure Database. Hydrogen atoms were added to all polar residues of the protein using AutoDock Vina, and the resulting PDBQT files were saved in the macromolecule directory. The docking site on the protein target was defined using a grid box, with the grid center coordinates (*x*, *y*, and *z*) and grid box size detailed in Table S3. We used the maximum dimensions of the grid box to cover the complete macromolecule, enabling the exploration of all potential binding sites on the protein surface.

#### 2.3.7 Statistical Analysis

All the experiments were conducted in triplicate, and results were expressed as mean ± standard deviation. Analysis of variance (ANOVA) was conducted using SPSS (version 17, IBM, USA). Duncan's multiple range test was applied to compare means at a significance level of *p* < 0.05.

## 3 Results and Discussion

### 3.1 Anti‐CaC2O4·NH2O Crystallization Effect

As for any crystallization process, the experiment of in vitro CaC_2_O_4_ nH_2_O crystallization was based on two‐phase phenomena of nucleation and aggregation. To follow these phases, the change in optical density in control and all test samples was recorded at an interval of 10 s for 15 min. In control, optical density increased up to 1.2 in 5 min, which indicated the progress in nucleation process and then decreased correspondingly due to aggregation in the remaining 10 min. In all test samples, the maximum optical density recorded was in the range of 0.35–0.5 in 5 min depending on the concentration of Exs and Frs, which shows that addition of any Ex/Fr inhibited CaC_2_O_4_·nH_2_O crystallization in a dose‐dependent manner. Similarly, decrease in nucleation and aggregation slopes was significant in all (*p* < 0.05) compared to control except where the %Ni and %Ai were <20%. All Exs and Frs showed >50% inhibition in nucleation and aggregation at 4 mg/mL except FFAFr, BBAFr, and FFNFr (Table 1).

**TABLE 1:** Nucleation and aggregation inhibition in the presence of extracts and fractions derived from sumac, pomegranate peel, almond leaves, falsa, and banana bracts.

	Reduction in nucleation (%)			Reduction in aggregation (%)		
	Sumac fruit					
Level (mg/mL)	Crude	Anthocyanin	Non‐anthocyanin	Crude	Anthocyanin	Non‐anthocyanin
**4**	90.14 + 0.89^[a]^	86.06 + 2.22^[a]^	87.34 + 3.25^[a]^	94.34 + 2.88^[1]^	90.31 + 2.98^[1]^	94.36 + 2.22^[1]^
**2**	76.07 + 4.10^[a]^	49.54 + 1.52^[b]^	57.01 + 3.29^[b]^	79.93 + 0.83^[1]^	61.29 + 1.61^[2]^	67.55 + 1.71^[2]^
**1**	54.51 + 1.45^[a]^	36.95 + 0.69^[c]^	41.06 + 2.33^[b]^	59.06 + 2.24^[1]^	47.47 + 3.96^[3]^	53.14 + 4.40^[2]^
	**Pomegranate peal**					
**4**	81.71 + 2.83^[a]^	75.50 + 1.07^[a]^	79.48 + 2.67^[a]^	90.02 + 1.79^[1]^	80.36 + 1.28^[2]^	86.01 + 2.05^[2]^
**2**	68.76 + 1.06^[a]^	40.09 + 2.63^[c]^	51.32 + 3.07^[b]^	73.57 + 4.16^[1]^	50.21 + 1.46^[2]^	62.90 + 2.87^[2]^
**1**	41.64 + 2.68^[a]^	34.19 + 1.42^[c]^	42.84 + 3.05^[a]^	52.74 + 3.20^[1]^	39.43 + 4.15^[2]^	41.11 + 2.63^[2]^
	**Indian almon leaves**					
**4**	62.13 + 1.71^[a]^	49.39 + 3.55^[c]^	55.26 + 2.83^[b]^	72.93 + 2.66^[1]^	51.70 + 3.16^[3]^	62.43 + 3.32^[2]^
**2**	47.24 + 3.18^[a]^	30.10 + 3.03^[c]^	39.31 + 1.97^[b]^	39.03 + 2.09^[2]^	40.62 + 3.56^[2]^	42.79 + 2.76^[1]^
**1**	30.68 + 3.45^[a]^	19.23 + 2.49^[b]^	21.39 + 1.26^[b]^	21.83 + 4.51^[2]^	23.93 + 2.45^[2]^	30.43 + 2.86^[1]^
	**Falsa fruit**					
**4**	92.20 + 3.58^[a]^	30.81 + 2.86^[c]^	49.87 + 0.25^[b]^	95.90 + 1.46^[1]^	41.37 + 2.64^[3]^	46.73 + 2.38^[2]^
**2**	80.37 + 2.15^[a]^	20.01 + 2.18^[c]^	36.97 + 2.61^[b]^	86.88 + 1.53^[1]^	14.10 + 2.57^[3]^	29.64 + 2.82^[2]^
**1**	68.79 + 1.87^[a]^	10.87 + 1.94^[c]^	17.98 + 1.83^[b]^	73.05 + 4.37^[1]^	17.29 + 4.38^[2]^	16.12 + 3.37^[2]^
	**Banana bract**					
**4**	88.91 + 1.31^[a]^	43.57 + 4.11^[c]^	62.24 + 2.71^[b]^	79.82 + 4.13 ^[1]^	51.23 + 1.27^[2]^	74.13 + 2.51^[1]^
**2**	68.55 + 4.16^[a]^	27.91 + 2.97^[c]^	54.05 + 3.49^[b]^	62.54 + 3.08^[1]^	37.90 + 0.58^[2]^	60.57 + 3.81^[1]^
**1**	54.71 + 2.74^[a]^	13.60 + 3.06^[c]^	36.70 + 3.02^[b]^	45.31 + 2.81^[1]^	20.85 + 3.49^[2]^	27.98 + 4.12^[2]^

*Note*: Different superscript alphabets (for nucleation) and numbers (for aggregation) in a column indicate statistically significant differences at *p* ≤ 0.05.

At a given concentration, the order of anti‐crystallization activity was found to be Ex > NFr > AFr. Another trend followed by most Exs and Frs was relatively higher %Ai than %Ni at all concentrations (Table 1). Anti‐crystallization effect of polyphenols, in general, lies in their ability to form soluble anionic species that facilitate their adsorption on the surface of crystals. Once adsorbed on surface, these anions block the active sites of crystal for further attachments and layering and ultimately reduce the size and aggregation of the crystals. In addition, their adsorption on the surfaces results in alteration of electrical charges that promotes attraction between the atoms on the surface and the ions present in solution [49]. The differences in anti‐crystallization activities of Exs, AFrs, and NFrs lie in their chemical composition. The reason for the highest anti‐crystallization activity of Exs can be attributed to the presence of organic acids in addition to polyphenols. Citrate, for example, lowers Ca^2+^ concentration and inhibits precipitation of calcium salts [50]. Moreover, malic acid, one of the main determinants of acidity in sumac [51], falsa [52], and pomegranate peel [53], is known to increase the citrate excretion through systematic alkalinization, which, in turn, favors Ca‐citrate complexation and reduces Ca^2+^ concentration. However, the potential of polyphenols alone as inhibitors is also strongly manifested in almost all AFrs and NFrs; NFrs suppressed CaC_2_O_4_·nH_2_O crystallization more effectively than AFrs at all concentrations but with few exceptions. For most anthocyanins, hydration is thermodynamically more favorable in the presence of water than proton transfer [54]. In addition, molecules such as gallic acid have four potential acidic protons with pK*_a_* values of 4.0 associated with their carboxylic group and pK*_a_* 8.7, 11.4, and >13 owing to their phenolic groups [55]. As the pK*_a_* value is 4.0, the anionic form of gallic acid dominates (>99.5%) in neutral solutions. Therefore, NFrs containing gallates, tannins, and phenolic acids may have greater capacity to donate protons and complex with Ca^2+^. Compared to ALEx and BBEx, the extracts SFEx, PPEx, and FFEx were found to have more pronounced effect on %Ni and % Ai at all concentrations (%Ni: 90, 82, 92; %Ai: 95, 90, 95, respectively, at 4 mg/mL), most probably due to the contribution of citric and malic acids as discussed before. In previous study on anti‐crystallization effect of pomegranate peel on calcium oxalate [36], hydroalcoholic extract of pomegranate peel showed 95% and 98% inhibition against nucleation and 83% and 89% against aggregation at 1 and 2 g/L, respectively. Compared to these % inhibition values, we determined lower values for both % nucleation (69, 42) and % aggregation (74, 53) at these concentrations. The difference in values can be explained on the basis of difference in extraction procedure and composition of extract that was dominated by ellagitannins. Phytochemical screening and inhibition of crystallization by ethanolic extract of falsa leaves [35] showed the presence of tannins and flavonoids in the leaf extract with 43.05% inhibition of Ca_2_O_4_·H_2_O that is significantly less than the % inhibition shown by falsa fruit in our study. Fruits contain variety of anthocyanins and non‐anthocyanins along with organic acids, which justifies their higher inhibition activity compared to leaf. Similarly, compared to % inhibition of CaC2O4·nH2O crystallization by BBEx at 2 mg/mL (68%) [34], it showed lower inhibition activity by metholic extract of banana stem at the same concentration. The banana bract owns its color due to variety of anthocyanins such as delphinidin, pelargonidin, paeonidin, and malvidin. Besides anthocyanins, BBEx [56] contains several other flavonoid compounds, which accounts for its higher activity compared to banana stem. Among AFrs, SFAFr and PPAFr showed much higher inhibition (%Ni: 85, 76; %Ai: 90, 80, respectively, at 4 mg/mL) compared to others. Compositional analysis of FFEx by Koley et al. revealed the presence of 69.2% cyanidin‐3‐sambubioside, 18.3% delphinidin‐3‐glucoside [28], whereas in ALEx 87% cyanidin‐3‐glucoside [31] and in BBEx 80% cyanidin‐3‐rutinoside were reported [57]. Compared to these, SFEx and PPEx contain a variety of anthocyanins (Table S2), which could presumably offer greater hindrance to crystallization in synergism. Similarly, due to high gallate and phenolic acid contents, SFNFr, PPNFr, and BBNFr demonstrated higher anti‐crystallization effect (Table 1).

### 3.2 Microscopy of CaC_2_O_4_·nH_2_O Crystals

**FIGURE 1:**
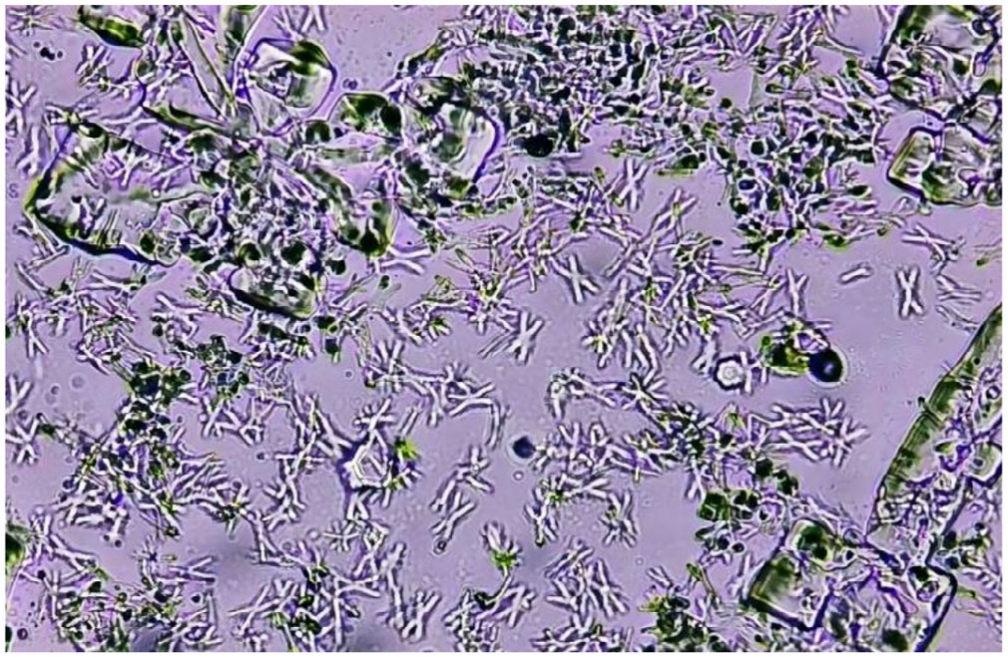
Photomicrograph showing twins of calcium oxalate monohydrate CaC_2_O_4_·H_2_O crystals and large aggregates in control at 400×.

CaC_2_O_4_·nH_2_O crystallization in the absence and presence of polyphenols was also analyzed by light microscopy, and pictures were captured and saved for multiple fields in the sampling area. For the control slide, the micrograph obtained after 24 h showed the presence of CaC_2_O_4_·H_2_O crystals in abundance and negligible number of CaC_2_O_4_·2H_2_O. Moreover, large aggregates of CaC_2_O_4_·H_2_O twins were seen in control (Figure 1).

**FIGURE 2:**
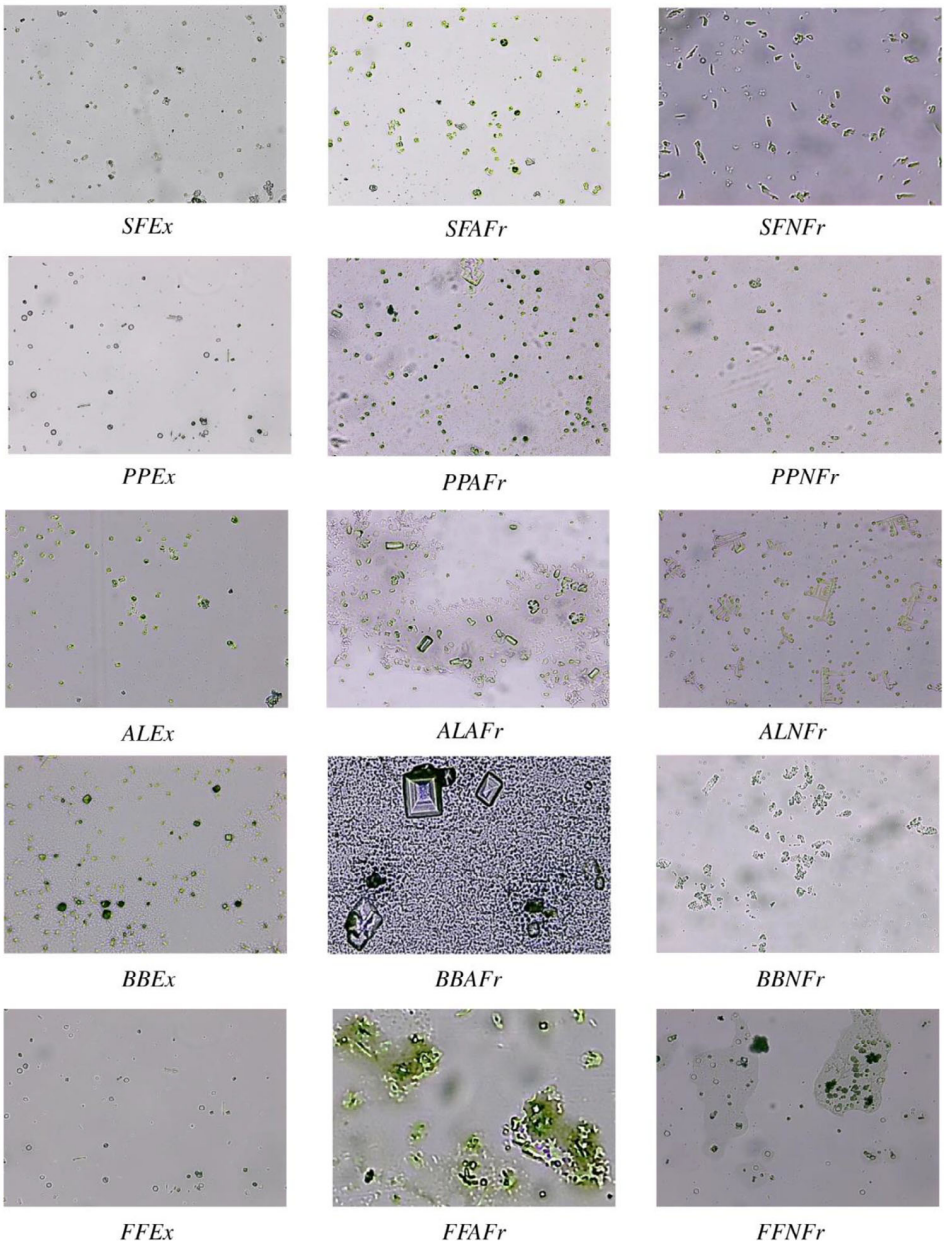
Photomicrograph showing different morphologies and size of calcium oxalate crystals and aggregates at 400× in the presence of extracts and fractions derived from sumac, pomegranate peel, almond leaves, falsa, and banana bracts. Extracts are generally more effective than fractions. AFr, anthocyanin fraction; AL, almond leaves; BB, banana bract; Ex, extract; FF, falsa fruit; NFr, non‐anthocyanin fraction; PP, pomegranate peel; SF, sumac fruit.

Compared to control, all test samples showed substantial decrease in number of CaC_2_O_4_·H_2_O crystals and aggregates (Figure 2); more aggregates and large crystals were seen in the presence of AFrs compared to NFrs and Exs. This is why least %Ni and %Ai were observed spectrophotometrically in the presence of AFrs. It has been established that CaC_2_O_4_·H_2_O crystals grow spirally in steps by incorporation of solute unless interrupted by inhibitors called step pinners and/or kink blockers [58 ^,^ 59]. Step pinners reduce the growth of crystal by adsorbing on the surface, whereas a kink blocker blocks solute in‐corporation into kinks. In previous studies, citrate was shown to play the role of step pinner as well as kink blocker. Further studies on synergistic effect of polyphenols (gallic acid, ellagic acid, pyrogallic acid) with citrate on suppression of CaC_2_O_4_·nH_2_O crystallization proved gallic acid as step pinner while ellagic acid and pyrogallic as both step pinners and kink blockers [8]. Thus, almost all Exs containing citrate/malate as well as gallic acid, ellagic acid, and pyrogallic acid showed maximum inhibition compared to control as the maximum crystal or aggregate size varied between 44 and 157 µm^2^ (SFEx 44, FFEx 54, PPEx 78, AlEx 144, BBEx 157 µm^2^) compared to control (∼6000 µm^2^) (Figure S2). Further, these crystals were mostly CaC_2_O_4_·2H_2_O, indicating the interference of Exs in conversion of CaC_2_O_4_·2H_2_O to CaC_2_O_4_·H_2_O. Next to Exs, maximum crystal size in case of NFrs was also significantly lower compared to control. For a given species, the order of crystal size was Ex < NFr < AFr, whereas among all the Exs and Frs, the order was SFEx < FFEx < SFNFr < PPEx < SFAFr < BBEx < PPNFr < PPAFr< ALEx < BBNFr < ALNFr < BBAFr < ALAFr < FFNFr < FFAFr, which was almost reverse of %N and %Ai. However, the least small CaC_2_O_4_·2H_2_O crystals were seen in the presence of Exs, and comparatively large crystals or aggregates formed in the presence of AFrs. In general, NFrs caused distortions in the shape of crystals with significant reduction in size.

### 3.3 Anti‐CHPD Crystallization Effect

**FIGURE 3:**
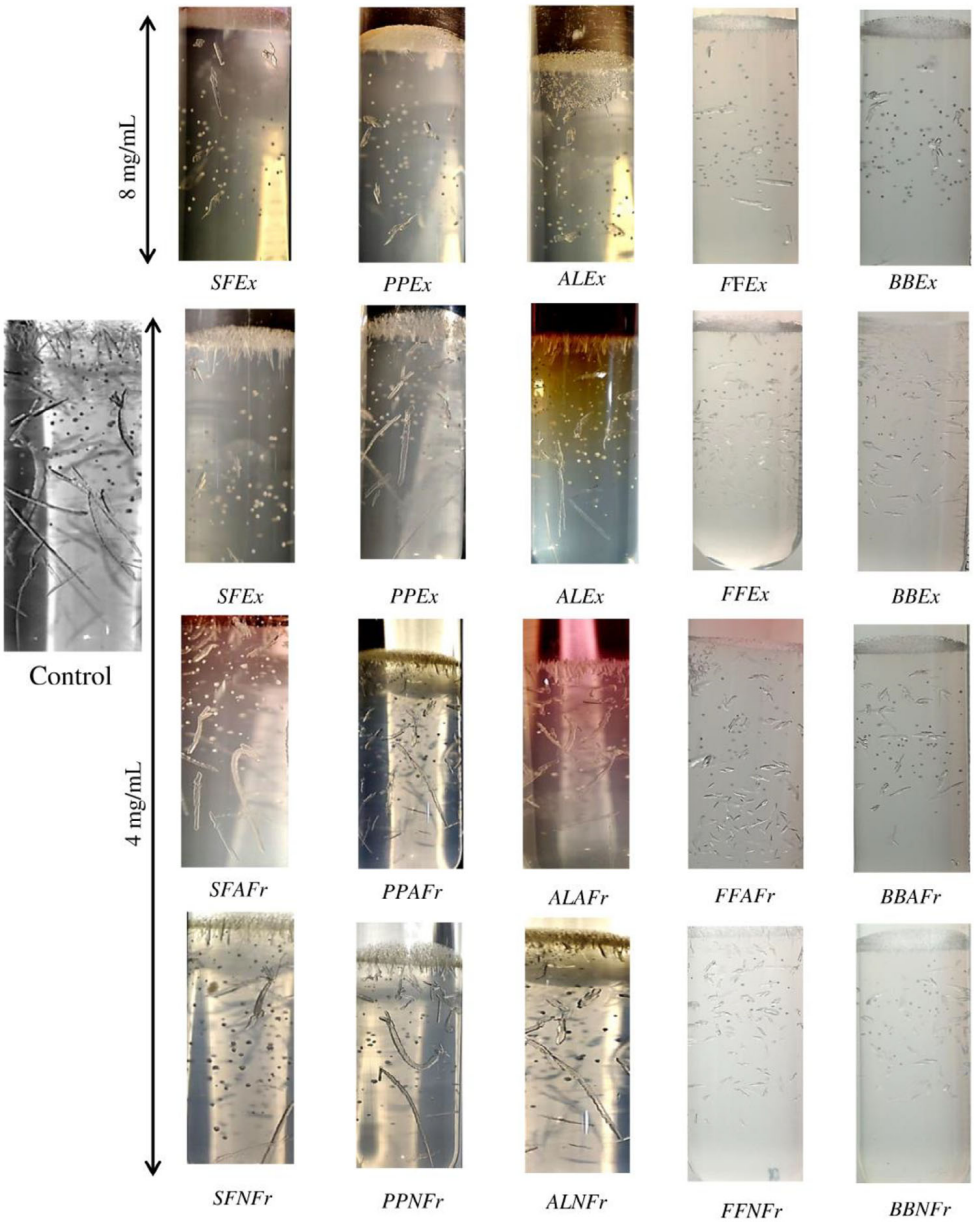
Effect of extracts (4 and 8 mg/mL) and fractions (4 mg/mL) on dissolution of brushite crystals. AFrs and NFrs of falsa and banana bracts promoted dissolution, whereas sumac, pomegranate, and almond fractions primarily reduced large crystal numbers, with inhibitory effects being more pronounced than dissolution. AL, almond leaves; AnFr, anthocyanin fraction; BB, banana bract; Ex, extract; FF, falsa fruit; NAnFr, non‐anthocyanin fraction; PP, pomegranate peel; SF, sumac fruit.

To determine the effect of Exs and Frs on the growth of brushite crystals, crystals were allowed to grow in the gel tubes for 5 days. Each day the length of crystals was measured; crystals acquired maximum length of 1.8 cm on Day 3 and 2.0 cm on Day 5. On Day 5, Exs and Frs were introduced in the gel tubes, and the length of crystals was monitored till eighth day. On eighth day, the number and size of crystals (<0.5 cm) reduced significantly in all Exs at 8 mg/mL compared to initial length of crystals (2.0 cm) in control (Figure 3). However, in the presence of Exs at 4 mg/mL, not only were the numbers of crystals comparatively higher, but also the maximum length measured was 1.0 cm in PPEx and AlEx that showed dose‐dependent growth inhibition. In AFrs and NFrs of falsa and banana bracts, pronounced dissolution effect was seen as the number of small broken crystals (<0.3 cm) increased, whereas in AFrs and NFrs of sumac, pomegranate, and almond, no significant change in maximum length was observed, yet the number of large crystals decreased compared to control (Figure 3).

In the case of sumac and pomegranate Exs and Frs, some crystals in the range of 0.5–0.75 cm formed at the interface, whereas very small (<1 mm) and few relatively large crystals (0.5 cm) grew in the gel; least inhibitory effect was observed in the presence of almond Exs and Frs. In comparison to control, where density of relatively large crystals (>0.7 mm) was observed, all Exs and Frs (except ALAFr and ALNFr) significantly affected the formation and growth of crystals. Further, compared to their dissolution effect, inhibitory effect of the Exs and Frs was more pronounced. This is perhaps due to the reason that dissolution requires adsorption of the inhibitor compounds on the surface of preformed crystals, which depends on the interaction of inhibitors with nuclei, whereas in inhibition experiments, polyphenolic inhibitors have easier access to calcium ions in solution and, thus, suppress the crystallization.

**FIGURE 4:**
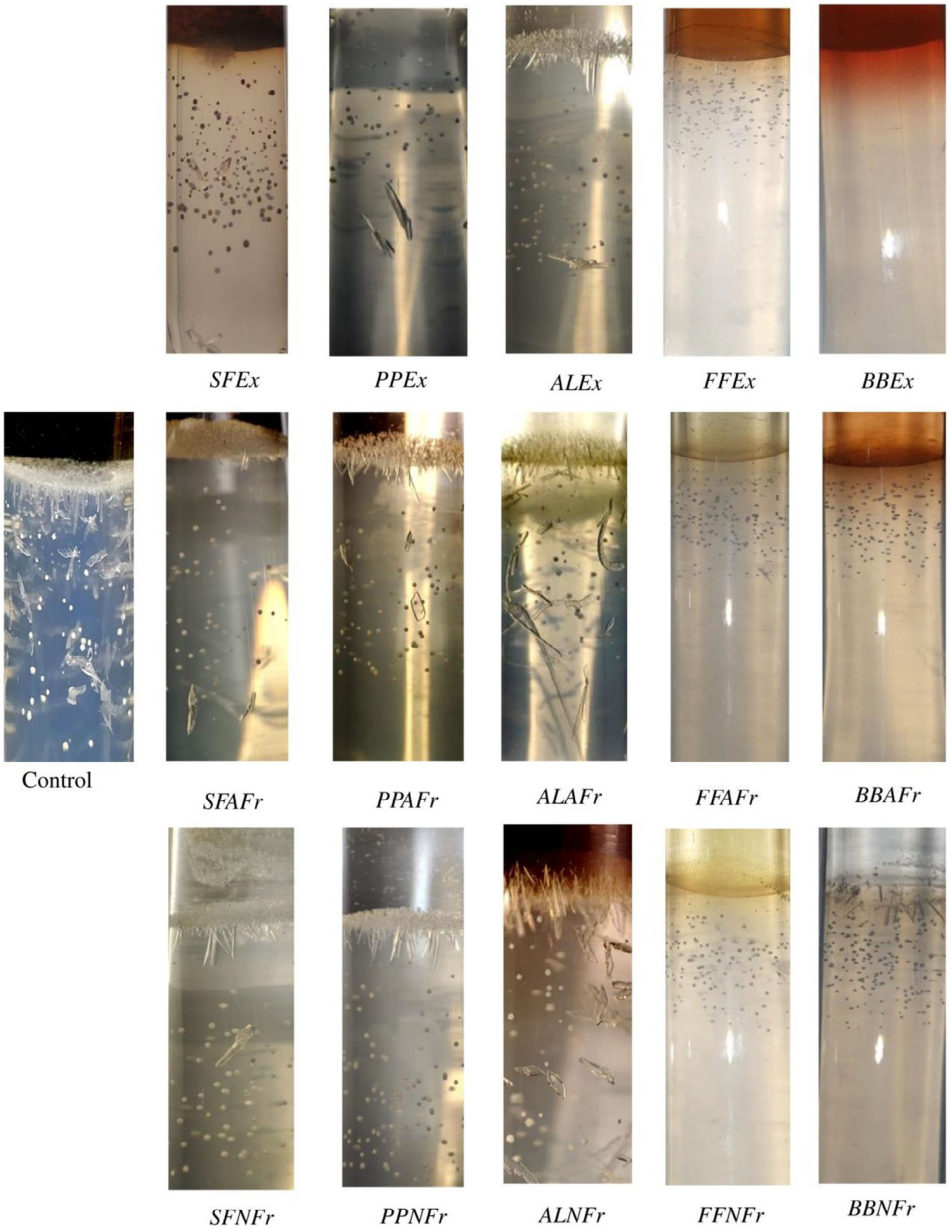
Inhibitory effect of extracts and fractions (4 mg/mL) on the formation of brushite crystals. BBEx showed complete inhibition, whereas very small (<1 mm) crystals formed in the presence of FFEx, FFAFr, FFNFr, BBAFr, and BBNFr. AL, almond leaves; AnFr, anthocyanin fraction; BB, banana bract; Ex, extract; FF, falsa fruit; NAnFr, non‐anthocyanin fraction; PP, pomegranate peel; SF, sumac fruit.

To determine the effect on the inhibition of crystal formation in the presence of Exs and Frs, calcium chloride and sample Exs/Frs were simultaneously introduced in the gel, and crystal formation was observed on Day 3. BBEx showed complete inhibition, whereas very small (<1 mm) crystals formed in the presence of FFEx, FFAFr, FFNFr, BBAFr, and BBNFr (Figure 4).

On the basis of their percentages in the Exs and Frs determined in previous studies, we selected 21 compounds (Table S2) for docking with 3ELB, IJ72, EFAY, UGGT2, and RIMBP3. The docking results showed that all ligands predominantly interacted through hydrogen bonding with acidic and basic amino acid residues (Table S4). Docking studies were performed by Aggarwal et al. [13] to predict the binding of EELB and IJF2 with CaC_2_O_4_·nH_2_O, also revealed the principal involvement of acidic and basic amino acids in interactions with CaC_2_O_4_·nH_2_O. Acidic amino acids were found to interact with calcium while basic with oxalate. This means that binding of polyphenolic ligands with acidic and basic amino acids of these proteins could effectively inhibit their binding with CaC_2_O_4_·nH_2_O. As NFrs of all polyphenolic sources showed greater in vitro anti‐crystallization effect on CaC_2_O_4_·nH_2_O and brushite crystals, likewise compounds such as sumaflavone (−11.5 to 9.0 kcal/mole), trigallic acid (−10.4 to −7.7 kcal/mole), punicalagin (−11.2 to −8.6 kcal/mole), and luteolin‐7‐(2apiosyl‐6‐malonyl glycoside) (−9.6 to −7.1 kcal/mole) present in the non‐anthocyanin fractions showed relatively higher binding energies on docking (Table S5, Figure S3).

Anthocyanin compounds, such as 7‐methyl‐cyanidin‐3‐galactoside, 7‐methyl‐cyanidin‐3‐(2″ galloyl) galactoside (comprising 53% and 35% of anthocyanins, respectively, in sumac), cyanidin‐3‐sambubioside (comprising 69.2% of anthocyanins in falsa), cyanidin‐3‐glucoside (comprising 50% and 87% of anthocyanins, respectively, in pomegranate peel and almond leaves), and cyanidin‐3‐rutinoside (80% of anthocyanins in banana bract), showed considerable binding potential as well (−9.7 to −6.6 kcal/mole) (Table S4, Figure S4). If anti‐CaC_2_O_4_·nH_2_O crystallization activities of the polyphenolic compounds are evaluated in terms of their binding ability with calcium ions, two important facts must be taken into consideration: First, those molecules that have two possible binding sites for calcium (a carboxylate group and a catechol group) such as gallic acid, ellagic acid, trigallic acid, *p*‐coumaric acid, and quinic acid are expected to bind more calcium, but at neutral pH, their catechol groups remain unionized, whereas carboxylate groups are deprotonated. Thus, carboxylate ions become the preferred binding sites for calcium ions [60]. Second, the decline in antioxidant activity of the phenolics in the presence of calcium indicates that catechol groups are also affected. Probably, the binding of calcium on the carboxylate group modifies the conformation of the aromatic part and thus the reactivity of its catechol groups.

## 4 Conclusion

This study demonstrates that polyphenol‐rich extracts (Exs), anthocyanin fractions (AFrs), and non‐anthocyanin fractions (NFrs) derived from sumac, pomegranate peel, almond leaves, falsa fruit, and banana bracts exhibit significant anti‐crystallization activity against calcium oxalate and brushite crystals. The order of anti‐crystallization efficacy was found to be Ex > NFr > AFr, with Exs showing the highest activity due to the synergistic effects of polyphenols and organic acids such as citrate and malate. These compounds inhibit crystal growth by adsorbing onto crystal surfaces, blocking active sites, and altering surface charges, thereby reducing crystal size and aggregation. NFrs, which are rich in gallates, tannins, and phenolic acids, demonstrated strong inhibition, likely due to their ability to donate protons and complex with Ca^2^⁺. AFrs, while less effective, still contributed to inhibition, particularly in sumac and pomegranate‐derived fractions. Polyphenols, in general, have the potential to inhibit the crystallization of calcium oxalate due to their ability to form anions that adsorb onto crystal surfaces and disrupt crystallization. However, their efficacy varies based on their structure, with non‐anthocyanin polyphenols proving more effective than anthocyanins from the selected plant sources. The study highlights that both anthocyanins and non‐anthocyanins inhibit stone formation through two distinct mechanisms: (1) interfering with the crystallization process and (2) binding with renal proteins to prevent crystal adhesion and further growth. This dual role of polyphenols suggests that falsa, pomegranate, almond leaves, and sumac could have potential applications in the prevention or treatment of renal stones. The findings underscore the therapeutic potential of polyphenolic compounds in preventing kidney stone formation, particularly calcium oxalate and brushite crystals. The study also emphasizes the importance of chemical composition, with organic acids and polyphenols playing critical roles in anti‐crystallization activity. However, the variability in inhibition efficacy across different sources and extraction methods indicates the need for further optimization of extraction protocols and compositional analysis. Future research should explore the therapeutic applications of these extracts, particularly in developing nutraceuticals or pharmaceuticals for individuals prone to kidney stone formation. Investigating the synergistic interactions between polyphenols and organic acids could further enhance anti‐crystallization efficacy. Mechanistic studies are also needed to elucidate the roles of step pinners and kink blockers in crystal inhibition.

Despite these promising findings, the study has several limitations. Differences in extraction procedures and plant sources may affect the consistency and efficacy of the extracts, limiting generalizability. The in vitro nature of the study means the results may not fully translate to in vivo conditions, necessitating animal or clinical studies for validation. Although 21 compounds were analyzed, the full spectrum of bioactive components in the extracts and fractions remains unexplored, potentially overlooking other contributors to anti‐crystallization activity. Another limitation of this study is the lack of investigation into the dissolution potential of the selected extracts. Specifically, the research did not explore whether the polyphenol‐rich extracts, anthocyanin fractions, and non‐anthocyanin fractions could dissolve already formed stones. This aspect is critical for understanding the full therapeutic potential of these compounds, as the ability to not only prevent but also dissolve kidney stones would significantly enhance their clinical applicability. Addressing these limitations will be crucial for translating these findings into effective treatments for kidney stone prevention.

## Author Contributions

**Mudassir Nazir**: methodology, software, formal analysis, data curation, writing – original draft preparation, investigation, validation. **Muhammad Abdul Haq**: conceptualization, methodology, software, validation, formal analysis, investigation, resources, data curation, writing – original draft preparation, writing – review and editing, visualization, supervision, project administration. **Syeda Moahida Batool Sherazi**: methodology, software, formal analysis, data curation, writing – original draft preparation, validation. **Shahina Naz**: methodology, software, formal analysis, investigation, data curation, writing – original draft preparation, validation. **Lubna Mobin**: methodology, software, investigation, validation. **Alexandros Tsoupras**: methodology, software, investigation, writing – review and editing, validation. All authors have read and agreed to the published version of the manuscript.

## Conflicts of Interest

The authors declare no conflicts of interest.

## Supporting information



Supporting Information

## Data Availability

The authors have nothing to report.
