# Supplementary material for: Effects of Polyphenolic Extracts From Sumac, Pomegranate Peel, Indian Almond Leaves, Falsa, and Banana Bracts on Calcium Oxalate and Brushite Crystallization In Vitro
Source: Chem Biodivers. 2025 Apr 8;22(7):e202500023. doi: 10.1002/cbdv.202500023 (PMC12270373; doi:10.1002/cbdv.202500023)
Supplement: Supplementary file 1 — Supporting Information [file CBDV-22-e202500023-s001.docx]

**Supporting Information**

Supporting Methods:

**Total phenolic contents (TPC):**

For the determination TPC, Folin-Ciocalteu assay^[^[^1^](#_ENREF_1)^]^ was used. A volume of 300 μL of the sample was mixed with 160 μL of Folin–Ciocalteau reagent and 250 μL of distilled water. Following a 5-minute incubation, 300 μL of a 10% sodium carbonate solution was introduced into the mixture. The reaction was then allowed to proceed for 30 minutes at ambient temperature. The absorbance was subsequently recorded at 750 nm, and the results were reported as milligrams of gallic acid equivalents (GAE) per gram of the sample.

**Total anthocyanin content:**

The total anthocyanin contents samples were determined by the pH differential method^[^[^2^](#_ENREF_2)^]^. An appropriate amount of each sample was separately mixed with 0.025 M hydrochloric acid–potassium chloride buffer (pH 1) and 0.4 M sodium acetate buffer (pH 4.5) to achieve an absorbance reading between 0.2 and 1.4. The absorbance of each mixture was measured at 520 nm and 700 nm, and the total anthocyanin content was expressed as cyanidin-3-glucoside equivalents.

Anthocyanin pigment (mg/L) = 𝐴 × MW × DF × V × 1000 /𝑎 ×𝑙 ×𝑚

where A is the absorbance, MW is the molecular weight of cyanidin-3-glucosode (449.2 g/mol), DF is the dilution factor, V is the solvent volume (mL), a is the molar absorptivity (26,900 L.mol−1.cm−1), and l is the cell path length (1 cm).

**Supporting Tables**

**Table S1**. Yield of crude extract, total phenolic content, and total anthocyanin in crude extract.

| **Sample** | **Crude extract (g/100 dry material)** | **Total Phenol (g GAE/100g of Crude extract)** | **Total Anthocyanin (g as cyanidin 3 glucoside/100g of Crude extract)** |
| --- | --- | --- | --- |
| **Sumac** | 28.3+2.1 | 40.8+3.2 | 5.3+0.2 |
| **Pomegranate peel** | 21.4+1.2 | 20.2+1.5 | 10.4+1.7 |
| **Indian almond leaves** | 15.1+2.8 | 19.6+2.7 | 7.8+0.9 |
| **Falsa** | 38.9+3.7 | 16.8+1.6 | 13.4+1.6 |
| **Banana Bract** | 10.8+1.7 | 14.31+1.2 | 12.7+0.9 |

**Table S2**.Phenolic compounds identified in sumac fruit, falsa, pomegranate peel, red almond leaves and banana bract.

| **Anthocyanin** | **Non- Anthocyanins** |
| --- | --- |
| **Sumac^[^**[**^3^**](#_ENREF_3)**^,^** [**^4^**](#_ENREF_4)**^]^** | |
| 7-Methyl-cyanidin-3-galactoside 53%^[a]^  7-Methyl-cyanidin-3- (2”galloyl) galactoside 35%^[a]^  Cyanidin-3-glucoside 7.8%^[a]^  Cyanidin-3- (2”galloyl) galactoside 3.8%^[a]^  Delphinidin-3-glucoside 0.25%^[a]^ | Sumaflavone^[qnd]^  Trigallic acid^[qnd]^  Gallic acid^[qnd]^  Ellagic acid^[nd]^ |
| **Falsa^[^**[**^5^**](#_ENREF_5)**^]^** | |
| Cyanidin-3-sambubioside 69.2%^[a]^  Delphinidin-3-glucoside 18.3%^[a]^ | Quercetin-3-glycosyl xyloside 54.36%^[b]^  Luteolin-7-(2apiosyl-6-malonyl) glycoside 90%^[c]^ |
| **Pomegranate peel ^[^**[**^3^**](#_ENREF_3)**^,^** [**^6^**](#_ENREF_6)**^]^** | |
| Cyanidin-3-glucoside 49%^[a]^  Pelargonidin-3-glucoside 25%^[a]^  Cyanidin-3,5-diglucoside 12%^[a]^  Pelargonidin-3,5-diglucoside 6%^[a]^  Delphinidin-3-glucoside 5%^[a]^  Delphinidin-3,5-diglucoside 3%^[a]^ | Punicalagin 77%^[d]^  Catechin 15%^[d]^  Ellagic acid 3%^[d]^  Gallic acid 3%^[d]^ |
| **Indian almond leaves^[^**[**^7^**](#_ENREF_7)**^,^** [**^8^**](#_ENREF_8)**^]^** | |
| Cyanidin-3-glucoside 87%^[a]^ | Punicalagin (ca. 0.48% of the dry weight) |
| **Banana bract^[^**[**^9^**](#_ENREF_9)**^]^** | |
| Cyanidin-3-rutinoside 80%a | p-Coumaric acid 41ppm  Quinic acid 13ppm |

[a]: % of total anthocyanin content, qnd: quantity not determined, [b]: %of total flavonols (flavonols =44.36% of total phenols), [c]: %of total flavones (flavones = 23.56% of total phenols), [d]: % of total monophenols (monophenols =76% of total phenols).

**Table S3**.Gridcenter coordinates and grid box dimensions chosen for autodocking of the selected ligands with target proteins.

| **Proteins with their PDB IDs** | **Grid center x y z coordinates** | **Grid box dimensions** | |
| --- | --- | --- | --- |
| **Ethanolamine-phosphate cytidylyltransferase (3ELB)** | X= 54, y= 110, z=13 | | X= 39, y =79, z= 84 |
| **Macrophage-capping protein (IJ72)** | x=36, y= 109, z= 11 | | x= 41, y= 82, z= 79 |
| **Ras GTPase-activating-like protein (3FAY)** | x= 25, y= 25, z= 10 | | x= 73, y= 44, z= 93 |
| **UDP glucose: Glycoprotein glucosyltransferase 2 (Gene: UGGT2)** | x=4, y= 3, z= 2 | | x= 120, y= 92, z=178 |
| **RIMS-binding protein 3A (Gene: RIMBP3)** | x = 5.0, y= 0.7, z= 3 | | x=203, y= 150, z= 182 |

**TableS4.**AutoDock Vina results showing acidic and basic amino acids of the receptor proteins (UDP-glucose- glycoprotein glycosyltransferase 2 (3ELB); Macrophage-capping protein (IJ72); Ras GTPase-activating-like protein (3FAY); UDP glucose: Glycoprotein glucosyltransferase 2 (Gene: UGGT2); RIMS-binding protein 3A (Gene: RIMBP3) involved in interactions with selected polyphenolic ligands.

| **Ligand molecule** | **3ELB** | **IJ72** | **UGGT2** | **RIMBP3** | **3FAY** |
| --- | --- | --- | --- | --- | --- |
| **7-Methyl-cyanidin-3- (2”galloyl) galactoside** | Asp10, Asp14, His20, Lys48, Asp97 | Lys142, Asp237, Asp241, Lys242, Arg352 | Glu735, Asp923 | Arg 312, Arg621, Lys625, Glu878, Arg886 | Lys1185, Lys1239 |
| **7-Methyl-cyanidin-3-galactoside** | Asp14, His23, Lys53, His101, Glu105 | Glu27, Asp46 | Asp1333, Asp1335 | Arg293, Lys625 Glu878, Arg886 | Lys997, Lys1000, Asp1003, Arg1060 |
| **Catechin** | Lys53, His101, Glu105 | Arg25 | Glu235, Glu1305, Arg1306 | Glu878, Arg886 | None |
| **Cyanidin-3- (2”galloyl) galactoside** | Asp14, His20, Lys48 | Luy142, Asp157, Glu235, ASp237 | Arg772, Asp734, Glu735 | Arg347, Glu339, Lys625 Glu878 | Lus997, Lys1053, Arg1060 |
| **Cyanidin-3,5-diglucoside** | Asp10, His20 | Lys252, Glu274, Asp280 | Asp1466, Lys1477 | Glu339, Arg347, Lys625, His852, Glu854, Glu878, Arg886 | Lys997, Lys1000, Arg1060 |
| **Cyanidin-3-glucoside** | His20, His94, Arg122 | Lys252, Glu274, Asp280 | Lys725, Asp733, Glu735 | Arg173, Glu177, Arg312, Lys625, Glu878, Arg886 | Lys1053 |
| **Cyanidin-3-rutinoside** | Asp10, His39, Asp97, Lys121, Arg122 | Arg152, Asp237, Asp267 | Lys1314, Asp1333, Asp1335, Asp1427, Asp1466, Lys1477 | Glu177, Arg312, Glu315, Lys625, Glu878, Arg886 | Lys1000, ASp1003, Lys1053, Arg1060, Arg1194 |
| **Cyanidin-3-sambubioside** | His20, His94, Asp97, Lys121, Arg122 | Lys142, Arg152, Asp237, Asp241 | Lys725, Arg772, Glu795, Asp923 | Arg173, Glu 177, Arg 312, Lys625, Glu854, Glu856, Glu878, Arg886 | Lys997, Lys1053, Arg1060 |
| **Delphinidin-3,5-diglucoside** | His20, His39, His94, Asp97 | Glu151, Arg152, Asp157, Asp241, Lys242, Asp267 | Lys63, Asp99, Lys405, Arg411 | Arg293, Arg347, Lys625, His852, Glu878, Arg886 | Lys997, Lys1000, Lys1053, Arg1060, Arg1194 |
| **Delphinidin-3-glucoside** | His20, Arg122 | none | Glu58, Asp998, Lys1181, Asp1163 | Arg163, Lys625, Glu854, Glu878, Arg886 | None |
| **Ellagic acid** | His94 | Lys252 | Arg1295 | Arg130, Asp874 | Lys1185 |
| **Gallic acid** | His23, His101, Lys103 | Arg25 | Arg1306 | Glu854, Glu856, Arg885, Arg886 | Arg1306 |
| **Luteolin-7-(2apiosyl-6-malonyl glycoside)** | Asp14, Asp17, His23, Lys53, HIs101, Glu105 | Arg318 | Asp734, Glu735, Arg772, Lys902, Asp923 | Lys625, Arg886 | Lys1053, Arg1060, Lys1185 |
| **p-coumaric acid** | None | Arg25 | Asp1335 | Arg885, Arg886 | None |
| **Pelargonidin-3,5-diglucoside** | Asp10, His17, His39, Lys48, His94, Asp97, Arg122 | Asp157, Asp241 | Asp734 | Arg173, Arg312, Glu315, His852, Glu856, Asp870,Asp872, Glu873, Asp874, Glu878 | Lys1053, Arg1060 |
| **Pelargonidin-3-glucoside** | His17, Lys48, His94 | Arg152, Lys230, Glu235, Asp237, Lys242 | Asp733, Asp734 | Arg312, Lys625, Glu878 | Lys997, Lys1053, Arg1060, Arg1194 |
| **Punicalagin** | Lys48, His94, Asp97, Arg122 | Arg137, Asp168, Arg201, Arg318 | Lys169, Asp171, His172, Lys405 | Arg293, Arg347, Lys625, His852, Glu854, Asp874, Glu878 | Asp1003, Lys1053, Arg1060, Arg1194 |
| **Quercetin-3-glycosyl xyloside** | Asp14, His20, Arg122 | Lys142, Arg152, Asp157, Asp237, Asp267 | Lys725, Asp734, Arg772 | Glu854, Glu878, Arg886 | Lys725, Asp734, Arg772 |
| **Quinic acid** | His101 | Lys142, Arg152, Asp157, Asp163 | None | Glu633, Glu856, Arg886 | None |
| **Sumaflavone** | None | Lys252 | None | Arg293, Arg347, Lys625, His852, Glu854, Glu878, Arg886 | None |
| **Trigallic acid** | His17, His20, His94 | Arg25, Asp46, Glu122 | Lys239, Lys1314, Asp1333, Asp1466, Lys1477 | Glu878, Arg885, Arg886 | Lys239, ys1314, Asp1333, Asp1466, Lys1477 |

**TableS5:** Binding energies (kcal/mole) of the selected molecules docked with UDP-glucose- glycoprotein glycosyltransferase 2 (3ELB); Macrophage-capping protein (IJ72); Ras GTPase-activating-like protein (3FAY); UDP glucose: Glycoprotein glucosyltransferase 2 (Gene: UGGT2); RIMS-binding protein 3A (Gene: RIMBP3).

| **Ligand molecule** | **3ELB** | **IJ72** | **UGGT2** | **RIMBP3** | **3FAY** |
| --- | --- | --- | --- | --- | --- |
| **7-Methyl-cyanidin-3- (2”galloyl) galactoside** | -8.8 | -8.3 | -9.5 | -7.2 | -7.8 |
| **7-Methyl-cyanidin-3-galactoside** | -9.5 | -7.3 | -8.4 | -6.8 | -8.2 |
| **Catechin** | -8.3 | -7.5 | -8.6 | -5.7 | -6.9 |
| **Cyanidin-3- (2”galloyl) galactoside** | -8.1 | -7.7 | -9.7 | -6.7 | -7.4 |
| **Cyanidin-3,5-diglucoside** | -9.7 | -7.7 | -8.5 | -7.1 | -7.7 |
| **Cyanidin-3-glucoside** | -8 | -7.6 | -9.6 | -7.1 | -7.5 |
| **Cyanidin-3-rutinoside** | -9.7 | -8.2 | -8.7 | -7.1 | -8.5 |
| **Cyanidin-3-sambubioside** | -8.6 | -8.1 | -9.5 | -6.6 | -7.8 |
| **Delphinidin-3,5-diglucoside** | -9.2 | -8.2 | -8.1 | -7 | -7.7 |
| **Delphinidin-3-glucoside** | -8.2 | -7.7 | -7.6 | -6.6 | -7.4 |
| **Ellagic acid** | -8 | -6.7 | -8.9 | -6 | -8.1 |
| **Gallic acid** | -6.5 | -6.2 | -6.1 | -5.6 | -5.8 |
| **Luteolin-7-(2apiosyl-6-malonyl glycoside)** | -9.6 | -7.1 | -8.7 | -7.5 | -8.9 |
| **p-coumaric acid** | -6.8 | -6.5 | -6.4 | -5.7 | -6.3 |
| **Pelargonidin-3,5-diglucoside** | -9.2 | -7.5 | -8.7 | -6.9 | -7.6 |
| **Pelargonidin-3-glucoside** | -8.1 | -7.1 | -9.4 | -6.2 | -7.8 |
| **Punicalagin** | -11.2 | -8.6 | -10 | -6.6 | -9.9 |
| **Quercetin-3-glycosyl xyloside** | -8.7 | -8 | -9.3 | -6.8 | -8.2 |
| **Quinic acid** | -6.3 | -5.4 | -6.4 | -5.1 | -5.6 |
| **Sumaflavone** | -11.3 | -10.4 | -10.8 | -9 | -11.5 |
| **Trigallic acid** | -9.1 | -8.5 | -10.4 | -7.7 | -8.3 |

**Supporting Figures**


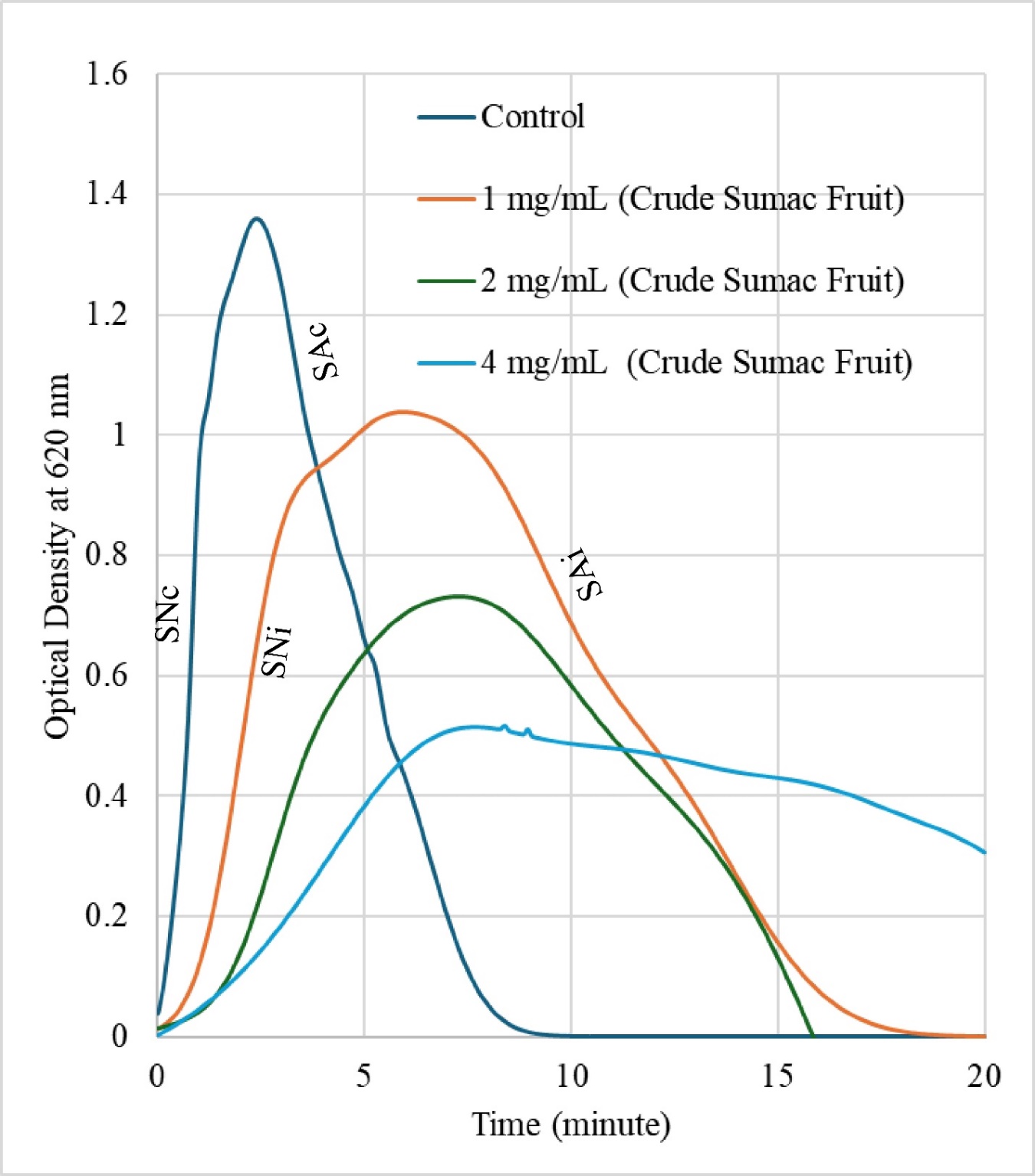


**Figure S1.**Representative time vs optical density curves of calcium oxalate crystallization in the presence and absence of polyphenolic extracts.


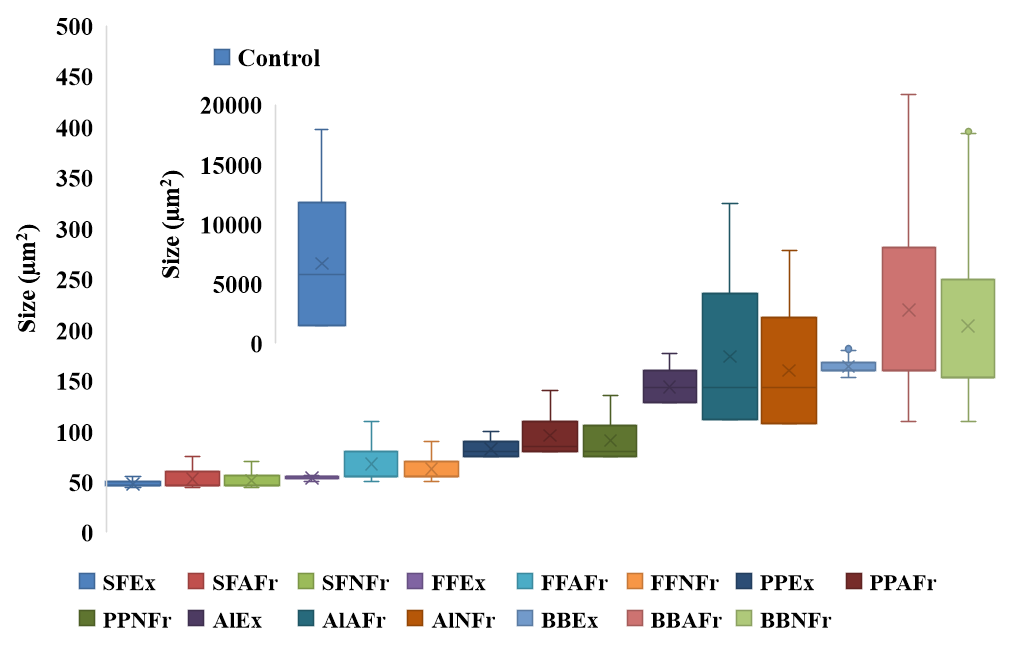


**Figure S2**. Size distribution of calcium oxalate crystals and aggregates formed in control and in presence of extracts and fractions derived from sumac, pomegranate peel, Almond leaves, Falsa, and banana bract. SF: Sumac fruit, PP: pomegranate peel, AL: Almond leaves, FF: Falsa fruit, BB: banana bract, Ex: extract, AFr: anthocyanin fraction, NFr: non-anthocyanin fraction.


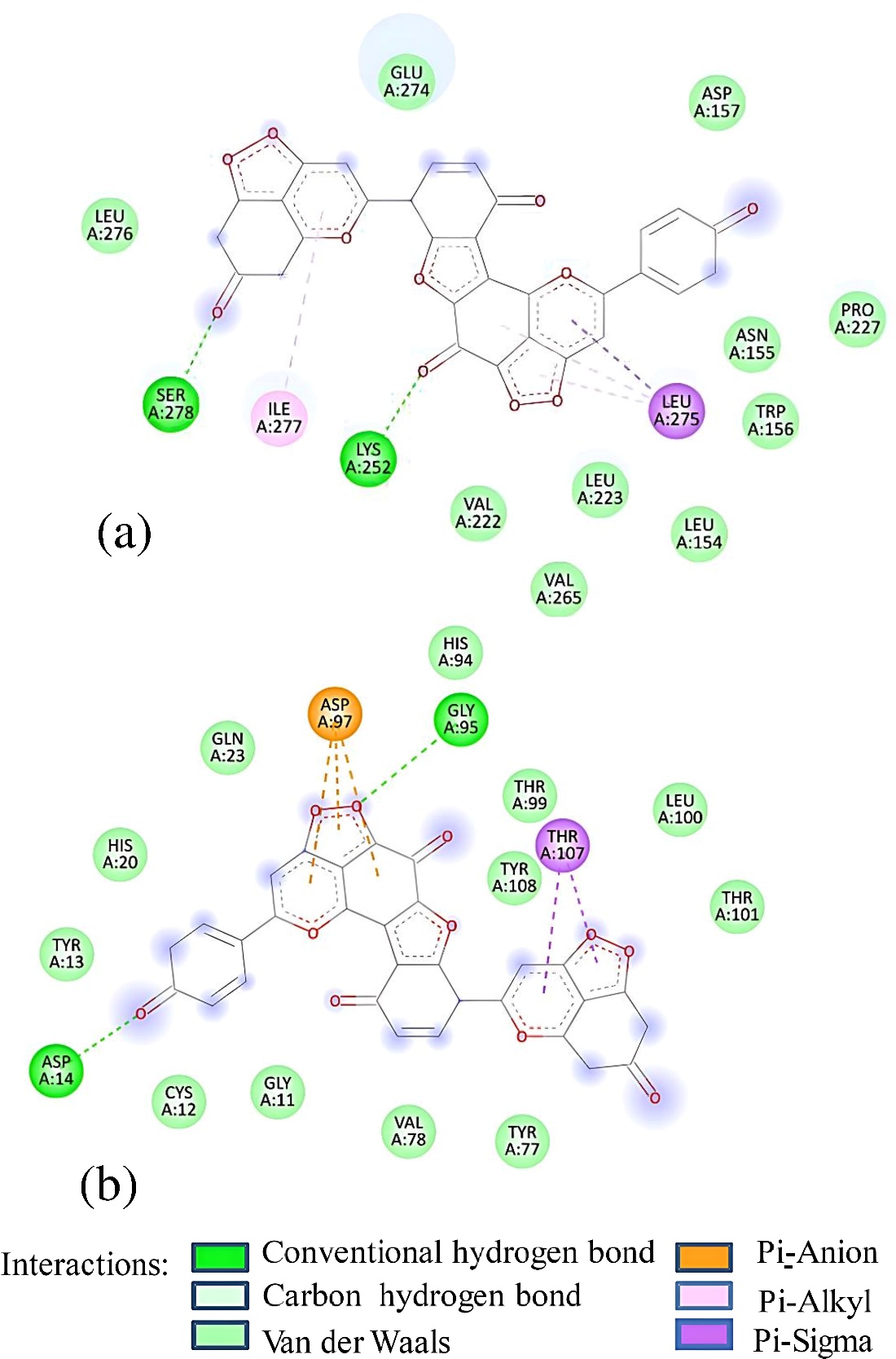


**Figure S3a-b**. 2D Diagram showing interaction of (a) sumaflavone with Macrophage-capping protein (PDB ID:IJ72); (b) sumaflavone with ethanolamine-phosphate cytidylyltransferase (PDB ID: 3ELB).


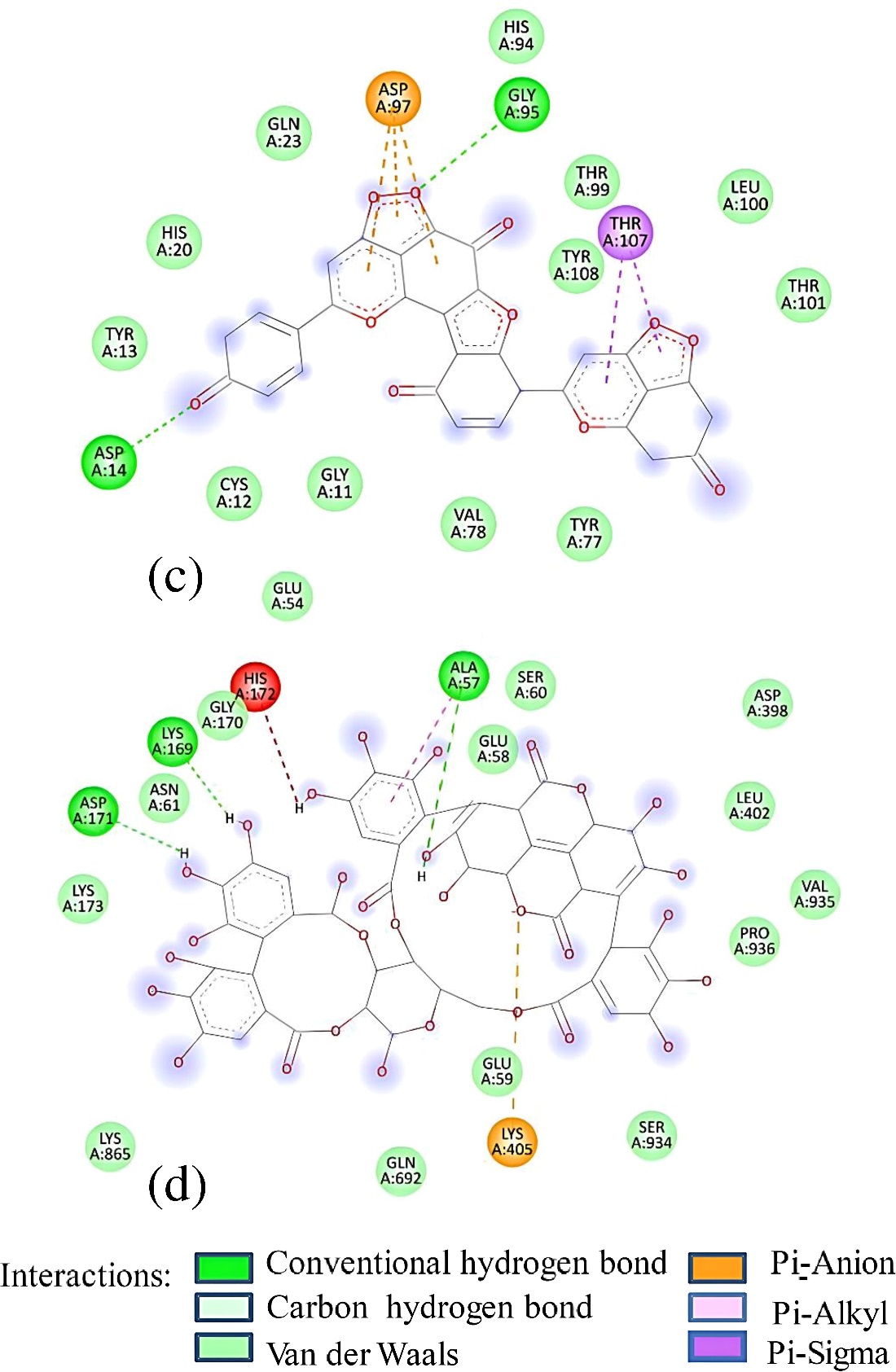


**Figure S3c-d.**2D Diagram showing interaction of (c) punicalagin with RIMS-binding protein 3A (Gene: RIMBP3); (d) punicalagin with UDP glucose: Glycoprotein glucosyltransferase 2 (Gene: UGGT2).


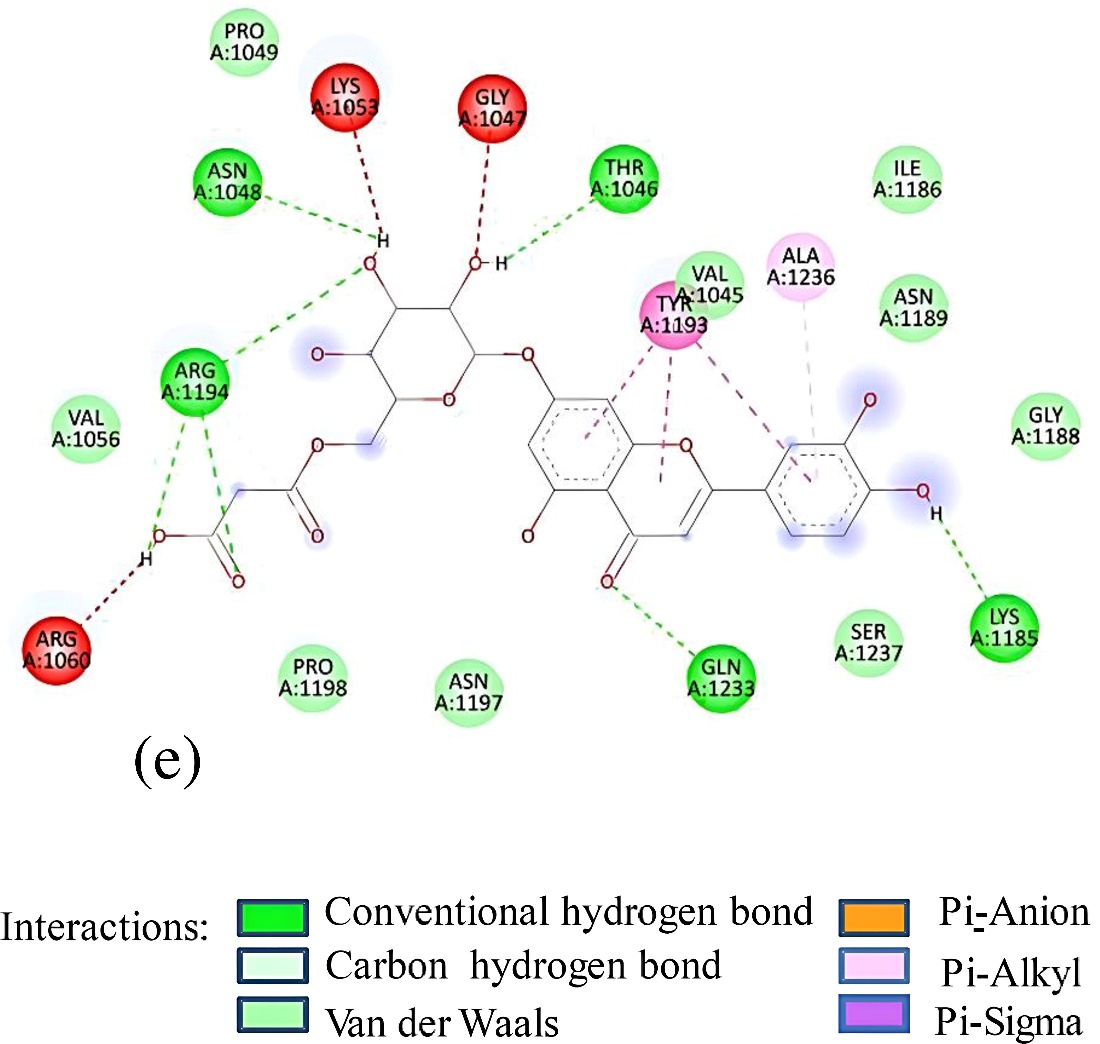


**Figure S3e**. 2D Diagram showing interaction of (e)Luteolin-7-(2apiosyl-6-malonyl) glycoside with Ras GTPase-activating-like protein (PDB ID:3FAY).


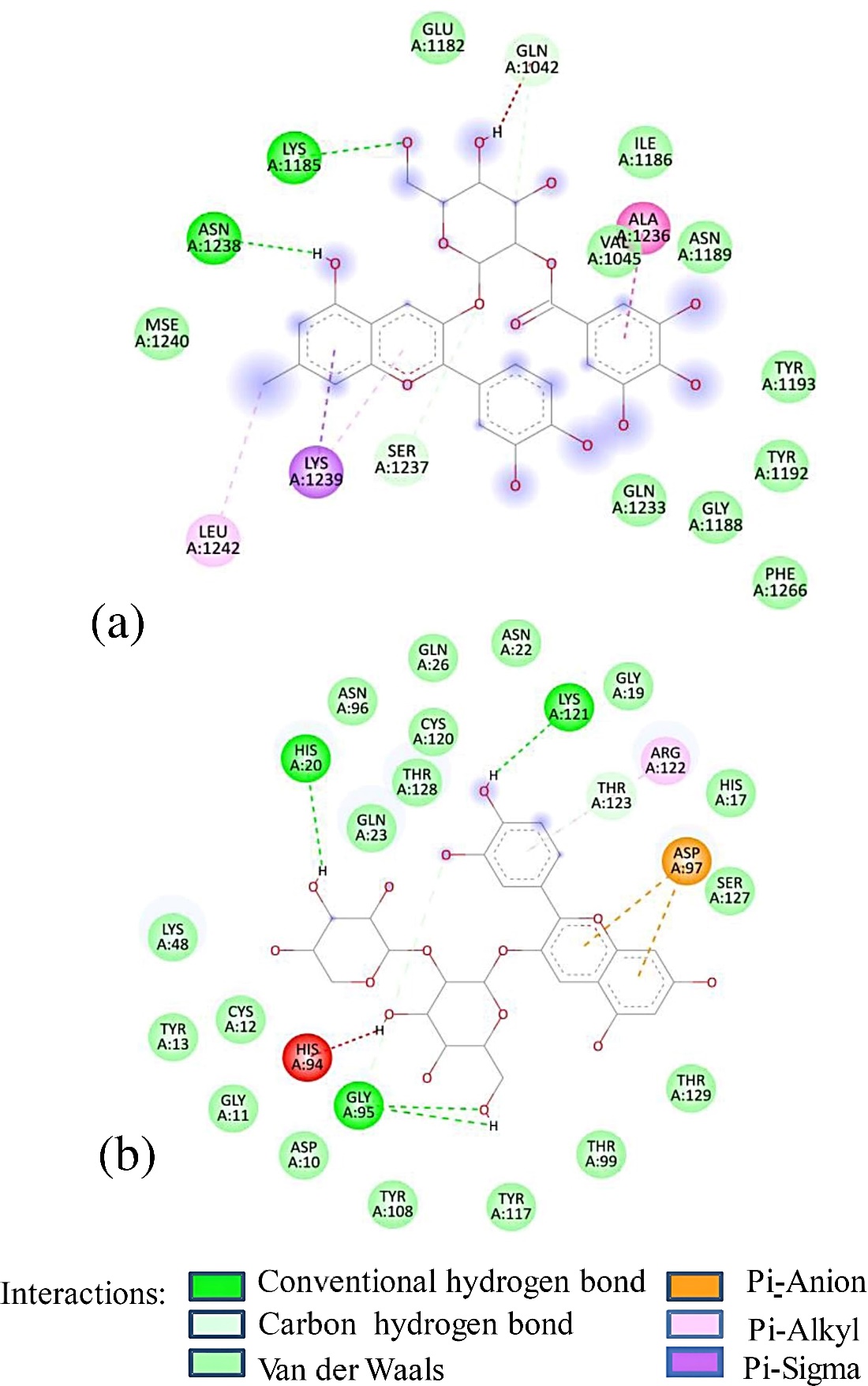


**Figure S4a-b.** 2D Diagram showing interaction of (a) 7-Methyl-cyanidin-3- (2״ galloyl) galactoside with Ras GTPase-activating-like protein (PDB ID:3FAY); (b) cyanidin-3-sambubioside with ethanolamine-phosphate cytidylyltransferase (PDB ID: 3ELB).


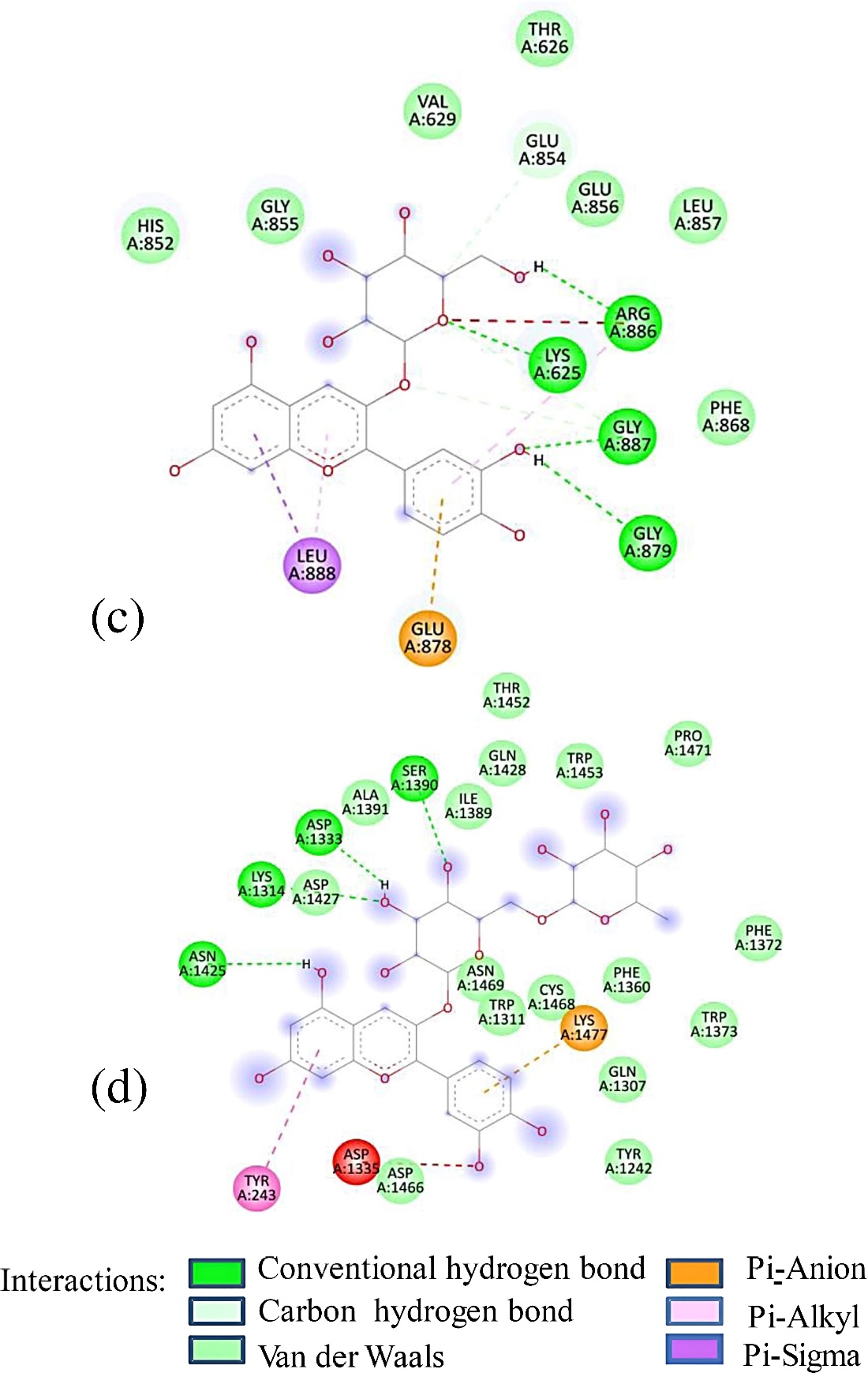


**Figure S4c-d.** 2D Diagram showing interaction of (c) cyanidin-3-glucoside with RIMS-binding protein 3A (Gene: RIMBP3); (d) cyanidin-3-rutinoside with UDP glucose: Glycoprotein glucosyltransferase 2 (Gene: UGGT2).

**References:**

[1] V. L. Singleton, J. A. Rossi, *Am. J. Enol. Vitic.* **1965**, *16*, 144-158.

[2] J. Lee, R. W. Durst, R. E. Wrolstad, Colleborators: T. Eisele, M. M. Giusti, J. Hach, H. Hofsommer, S. Koswig, D. A. Krueger, S. Kupina; S. K. Martin, B. K. Martinsen, T. C. Miller, F. Paquette, A. Ryabkova, G. Skrede, U. Trenn, J. D. Wightman, *J. AOAC Int.* **2005**, *88*, 1269-1278.

[3] F. V. Romeo, G. Ballistreri, S. Fabroni, S. Pangallo, M. G. Li Destri Nicosia, L. Schena, P. Rapisarda, *Molecules* **2015**, *20*, 11941-11958.

[4] M. Kosar, B. Bozan, F. Temelli, K. Baser, *Food Chem.* **2007**, *103*, 952-959.

[5] T. K. Koley, Z. Khan, D. Oulkar, B. Singh, B. Bhatt, K. Banerjee, *J. Food Sci.Technol.* **2020**, *57*, 606-616.

[6] J. Li, X. He, M. Li, W. Zhao, L. Liu, X. Kong, *Food Chem.* **2015**, *176*, 7-11.

[7] A. Anand, N. Divya, P. Kotti, *Phcog. Rev.* **2015**, *9*, 93.

[8] T. Tanaka, G. I. Nonaka, I. Nishioka, *Chem. Pharm. Bull.* **1986**, *34*, 1039-1049.

[9] E. A. Pazmiño-Durán, M. M. Giusti, R. E. Wrolstad, M. B. A. Glória, *Food Chem.* **2001**, *73*, 327-332.
